# Supplementary material for: Growth hormone releasing hormone signaling promotes Th17 cell differentiation and autoimmune inflammation
Source: Nat Commun. 2023 Jun 6;14:3298. doi: 10.1038/s41467-023-39023-1 (PMC10244428; doi:10.1038/s41467-023-39023-1)
Supplement: Supplementary file 1 — Supplementary Information [file 41467_2023_39023_MOESM1_ESM.pdf]

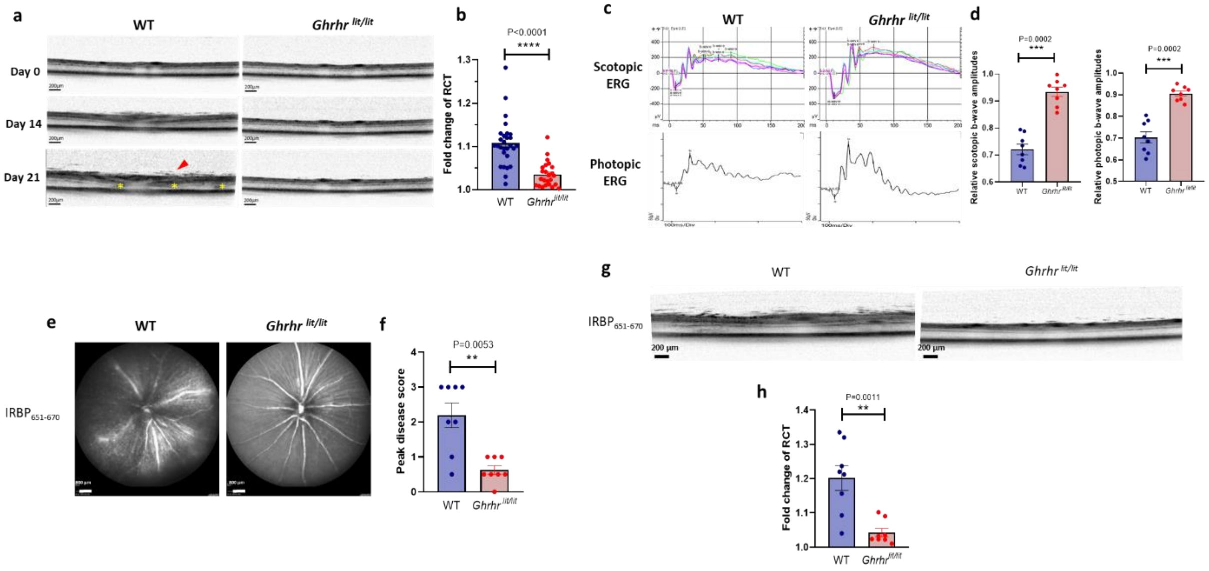

Supplementary Fig. 1. Deficiency of GHRH-R protects mice from autoimmune ocular inflammation.

(a-d) Age and gender matched WT mice (n=32) and *Ghrhr*$^{lit/lit}$ mice (n=28) were challenged with EAU and assessed by OCT and ERG at day 0, day 14 and day 21 after immunization. (a) Representative images by OCT at 3 time points. Eye-infiltrating cells (arrowhead) and retinal fold (asterisk) in the vitreous and retina are indicated. Scale bar: 200 μm. (b) Quantified fold change of RCT assessed by OCT at day 21 after immunization. (c) Representative scotopic ERG (top) under light intensity of 1 cd.s/m^2 and photopic ERG (bottom) under light intensity of 10 cd.s/m^2 at day 21 after immunization. (d) Quantified relative fold change of b-wave amplitude in scotopic and photopic ERG (n=8 per group). (e-h) Age and gender matched WT mice (n=8) and *Ghrhr*$^{lit/lit}$ mice (n=8) were immunized with IRBP$_{651-670}$ peptide, complete Freund's adjuvant (CFA) and pertussis toxin (PTX) to challenge with EAU and were followed 21 days. (e) Representative fundus images by cSLO after immunization. Scale bar: 800 μm. (f) Peak disease scores of uveitis throughout 21 days. (g) Representative images by OCT. Data are representation of at least two independent experiments. (h) Quantified fold change of RCT assessed by OCT on day 21 after immunization.

Data are presented as mean ± SEM. P-values were all determined by Mann-Whitney test. Statistical tests were all two-sided. **, *** and **** represent $P<0.01$, $P<0.001$ and $P<0.0001$ respectively.

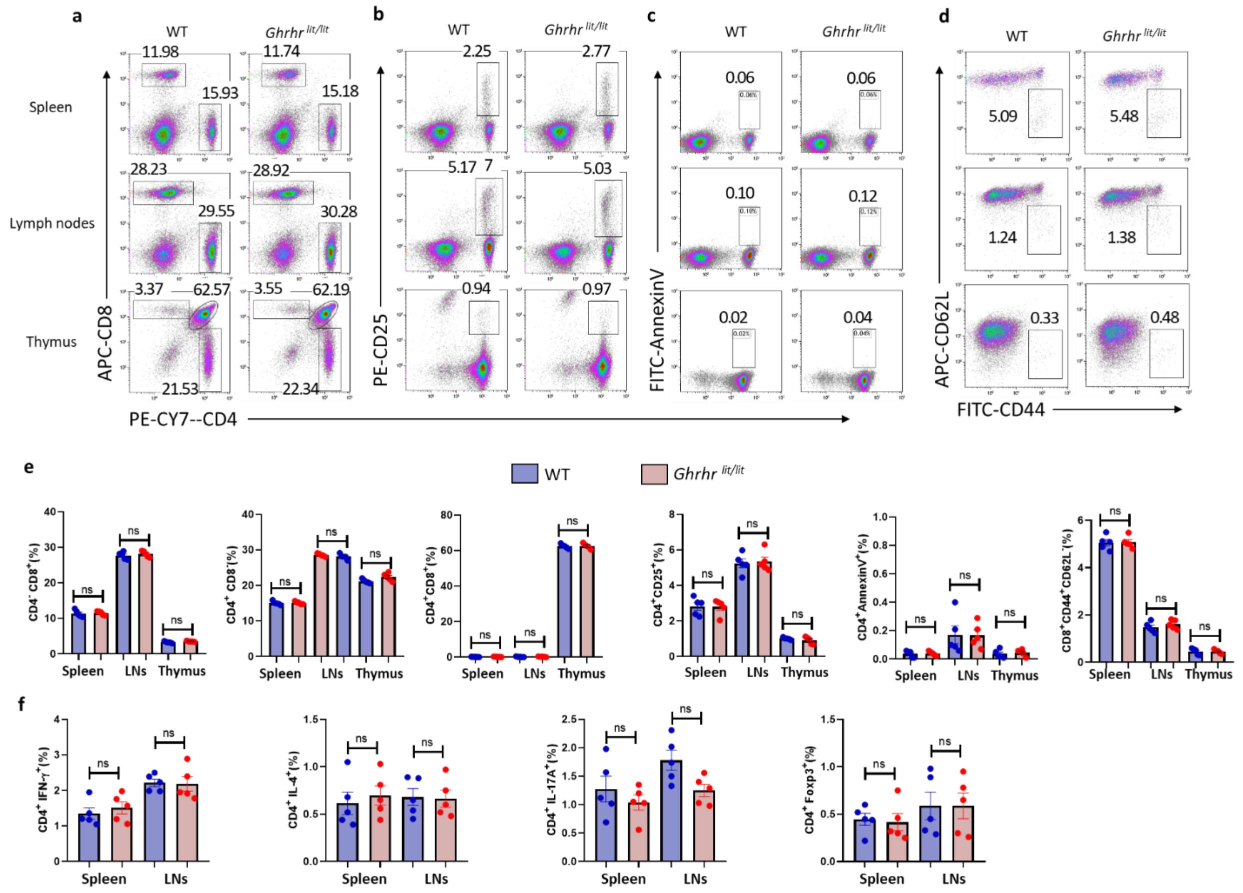

Supplementary Fig. 2. Deficiency of GHRH-R does not alter homeostasis of lymphocyte populations.

Age and gender matched WT mice and *Ghrhr*$^{lit/lit}$ mice were used. (a-d) Flow cytometry analysis of CD4, CD8, CD44, CD62L expression as well as apoptosis level in the lymphocytes from spleen, draining lymph nodes and thymus. (e) Quantified data depicted in (a-d). (f) Quantified expression of IFN-γ, IL-4, IL-17A and Foxp3 in the CD4$^+$ T cells isolated from spleen and draining lymph nodes. LNs: Lymph nodes. Data are representative of four independent experiments (n=5 per group). Data are presented as mean ± SEM. P-values were determined by Student's t-test. ns represents no significant difference. Statistical tests were all two-sided.

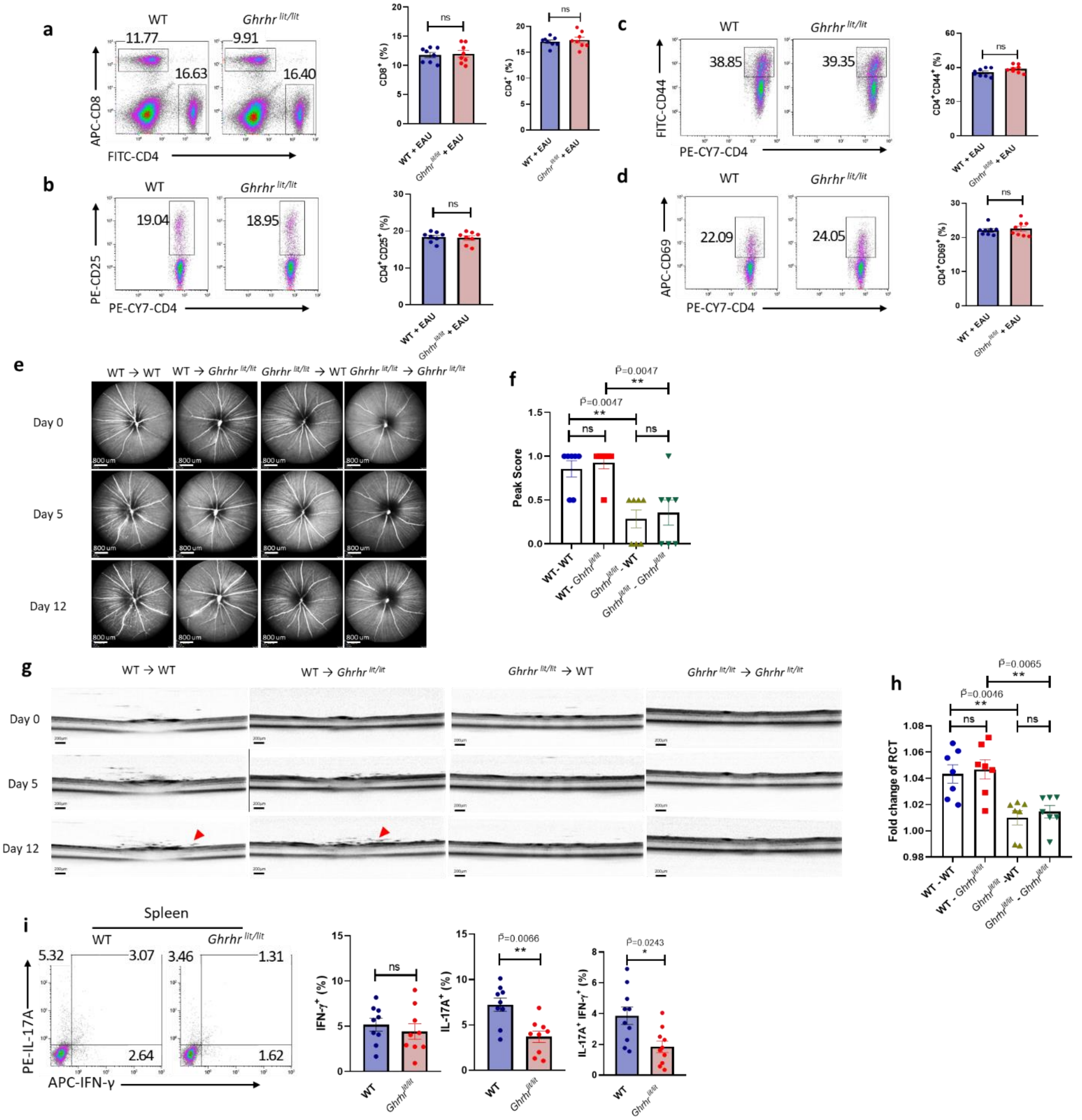

Supplementary Fig. 3. Deficiency of GHRH-R does not alter lymphocyte population in the autoimmune ocular uveitis.

(a-d) Lymphocytes were isolated from the eye-draining lymph nodes of IRBP-immunized WT mice and *Ghrhr*$^{lit/lit}$ mice. (a) Flow cytometry analysis of CD4 and CD8 population in the lymphocytes (n=5). (b-d) Flow cytometry analysis of CD25, CD44 and CD69 expression in the

$CD4^+$ T cells (n=5). (e) $CD4^+$ T cells from $IRBP_{1-20}$-immunized donor WT and *Ghrhr*$^{lit/lit}$ mice were cultured under Th17 cell-polarizing conditions (IL-6, TGF-β and IL-23) with $IRBP_{1-20}$ peptide for 3 days and adoptively transferred into naïve WT and *Ghrhr*$^{lit/lit}$ recipient mice. Representative fundus images by cSLO at 3 time points after adoptive transfer are shown. Scale bar: 600 μm. (f) The peak disease score of uveitis 12 days after adoptive transfer (n=7). The F value of one-way ANOVA test is 10, and the corresponding p-value is 0.0002. (g) Representative images by OCT at 3 time points after adoptive transfer. Scale bar: 200 μm. Eye-infiltrating cells (arrowhead) could be observed in the vitreous and retina. This experiment was repeated two times independently with similar results. (h) Fold change of retinal choroidal thickness assessed by OCT at day 12 after adoptive transfer (n=7). The F value of one-way ANOVA test is 9.363, and the corresponding p-value is 0.0003. (i) WT and *Ghrhr*$^{lit/lit}$ mice were challenged with EAU. After 21 days, cells were isolated from spleen. IFN-γ, and IL-17A expression in $CD4^+$ T cells (n=10). Data are presented as mean ± SEM. Two-sided student's t-test with Bonferroni correction (a-d and i) and one-way ANOVA followed by Bonferroni post hoc test (f and h). * and ** represent $\widetilde{P}<0.05$ and $\widetilde{P}<0.01$ respectively. ns represents no significant difference.

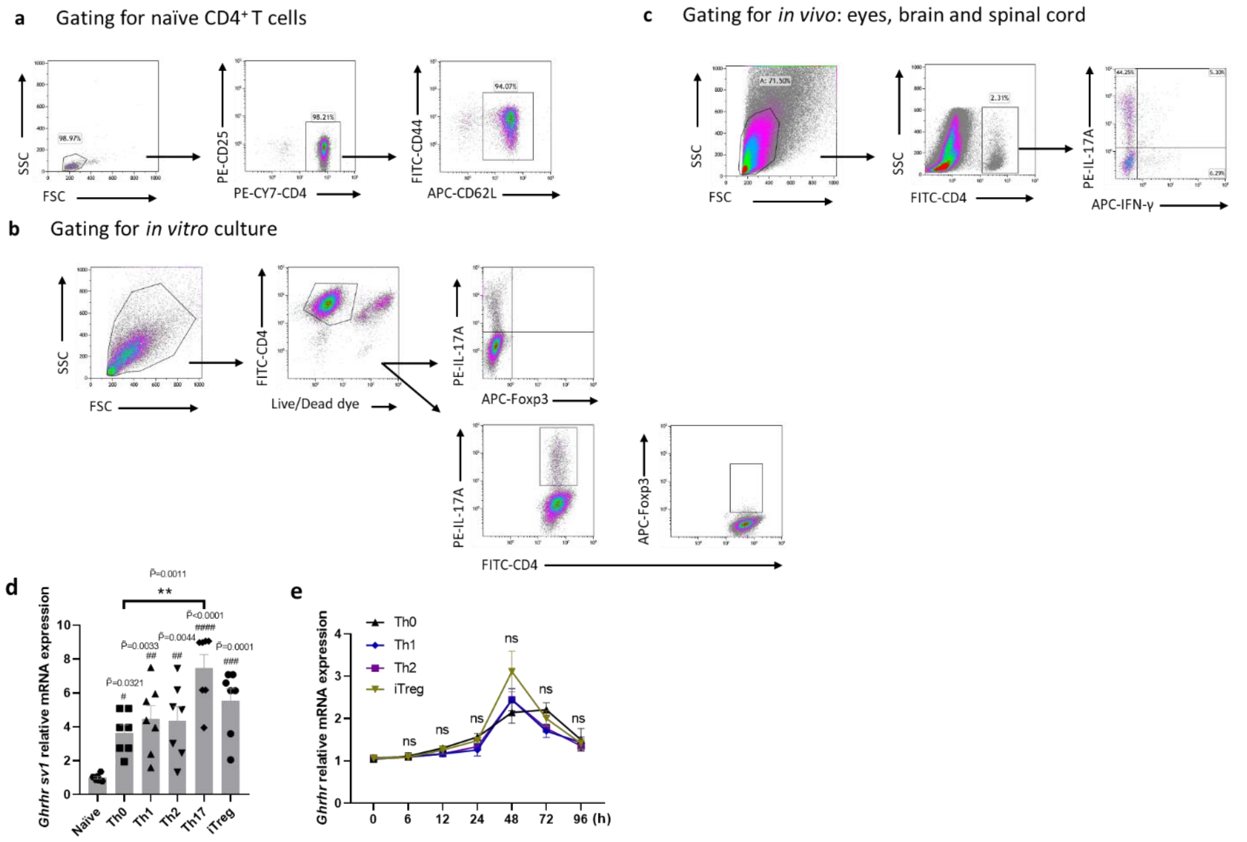

Supplementary Fig. 4. Gating strategy for the flow cytometry analysis

(a) Naïve CD4$^+$ T cells were purified from the spleen and the purity of naïve CD4$^+$ T cells were investigated by flow cytometry. (b) Gating strategy for the flow cytometry analysis of *in vitro* cultured T cells and (c) *in vivo* derived cells from the eyes, brain and spinal cord. (d-e) Naïve CD4$^+$ T cells were cultured in the stimulation with anti-CD3 and anti-CD28 antibodies under Th differentiation condition: None (Th0), IL-12 (Th1), IL-4 (Th2), IL-6 and TGF-β (Th17), TGF-β (iTreg). (d) At 48 hours after culture, *Ghrhr sv1* gene expression were assessed by RT-qPCR and normalized to the housekeeping gene *Gapdh*; fold change is relative to naïve controls (n=7 independent experiments). The F value of one-way ANOVA test is 10.4, and the corresponding p-value is less than 0.0001. (e) Naïve CD4$^+$ T cells were activated (Th0) and differentiated into Th1, Th2 and iTreg cells. *Ghrhr* gene expression was evaluated along the 7 time points throughout the

96 h differentiation (n=5 independent experiments). Data are representation of at least two independent experiments. Data are presented as mean ± SEM. Two-sided student's t-test with Bonferroni correction (e) and one-way ANOVA followed by Bonferroni post hoc test (d). #, ## and #### represent $\tilde{P}<0.05$, $\tilde{P}<0.01$and $\tilde{P}<0.0001$ when compared to naïve T cells. ** represents $\tilde{P}<0.01$. ns represents no significant difference.

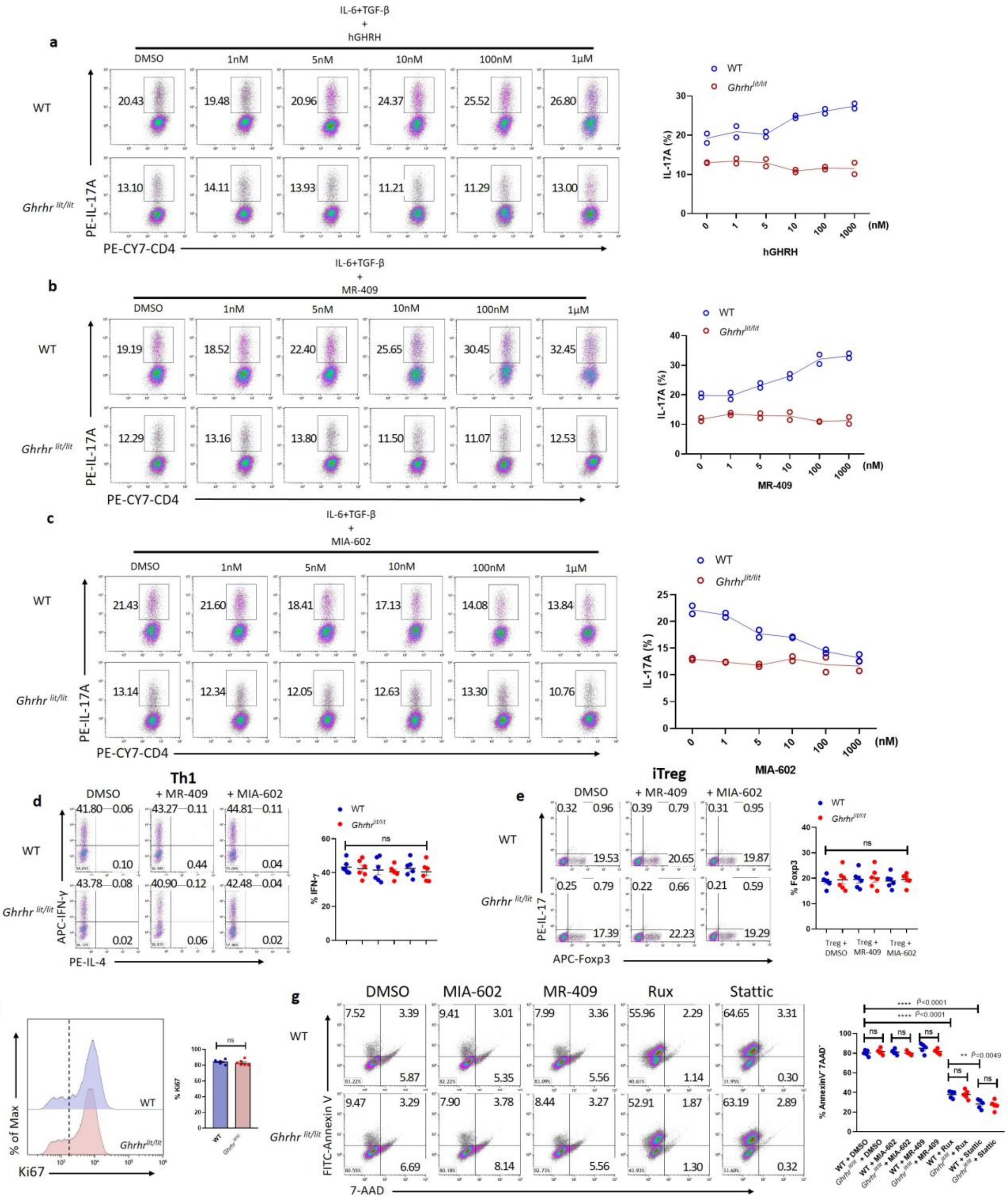

a
IL-6+TGF-β + hGHRH
DMSO 1nM 5nM 10nM 100nM 1μM
WT 20.43 19.48 20.96 24.37 25.52 26.80
Ghrhr lit/lit 13.10 14.11 13.93 11.21 11.29 13.00
PE-IL-17A
PE-CY7-CD4
WT
Ghrhr lit/lit
IL-17A (%)
hGHRH
(nM)
b
IL-6+TGF-β + MR-409
DMSO 1nM 5nM 10nM 100nM 1μM
WT 19.19 18.52 22.40 25.65 30.45 32.45
Ghrhr lit/lit 12.29 13.16 13.80 11.50 11.07 12.53
PE-IL-17A
PE-CY7-CD4
MR-409
c
IL-6+TGF-β + MIA-602
DMSO 1nM 5nM 10nM 100nM 1μM
WT 21.43 21.60 18.41 17.13 14.08 13.84
Ghrhr lit/lit 13.14 12.34 12.05 12.63 13.30 10.76
PE-IL-17A
PE-CY7-CD4
MIA-602
d
Th1
DMSO + MR-409 + MIA-602
WT 41.80 0.06 0.10 43.27 0.11 0.44 44.81 0.11 0.04
Ghrhr lit/lit 43.78 0.08 0.02 40.90 0.12 0.06 42.48 0.04 0.02
APC-IFN-γ
PE-IL-4
% IFN-γ
ns
e
iTreg
DMSO + MR-409 + MIA-602
WT 0.32 0.96 19.53 0.39 0.79 20.65 0.31 0.95 19.87
Ghrhr lit/lit 0.25 0.79 17.39 0.22 0.66 22.23 0.21 0.59 19.29
PE-IL-17
APC-Foxp3
% Foxp3
Treg + DMSO
Treg + MR-409
Treg + MIA-602
f
% of Max
Ki67
% Ki67
g
DMSO MIA-602 MR-409 Rux Stattic
WT 7.52 3.39 5.87 9.41 3.01 5.35 7.99 3.36 5.56 55.96 2.29 1.14 64.65 3.31 0.30
Ghrhr lit/lit 9.47 3.29 6.69 7.90 3.78 8.14 8.44 3.27 5.56 52.91 1.87 1.30 63.19 2.89 0.32
FITC-Annexin V
7-AAD
% AnnexinV⁻ 7AAD⁻
**** P<0.0001
** P=0.0049
WT + DMSO
Ghrhr lit/lit + DMSO
WT + MIA-602
Ghrhr lit/lit + MIA-602
WT + MR-409
Ghrhr lit/lit + MR-409
WT + Rux
Ghrhr lit/lit + Rux
WT + Stattic
Ghrhr lit/lit + Stattic

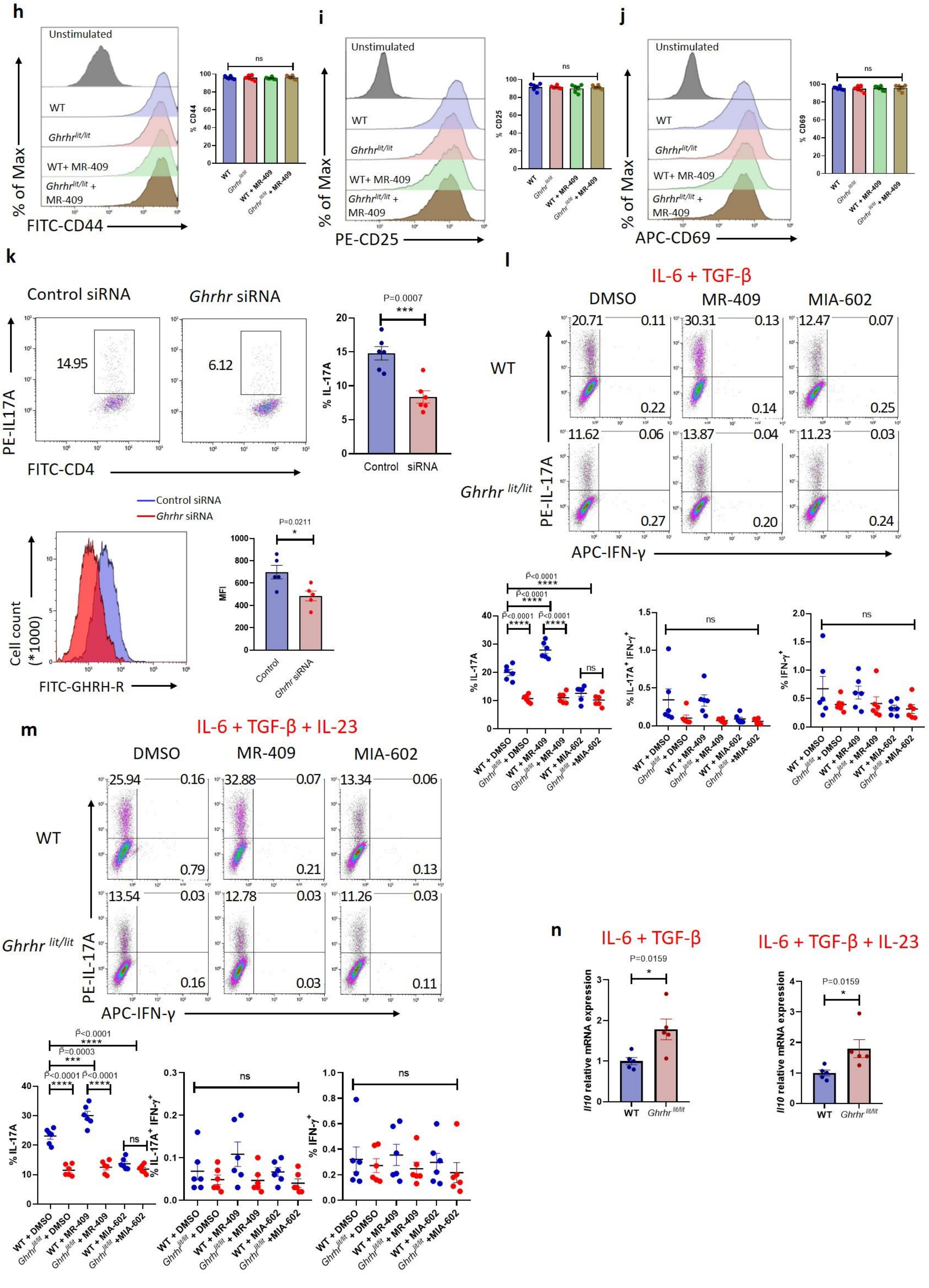
h
Unstimulated
WT
Ghrhr lit/lit
WT+ MR-409
Ghrhr lit/lit + MR-409
% of Max
FITC-CD44
% CD44
ns
i
PE-CD25
% CD25
j
APC-CD69
% CD69
k
Control siRNA
Ghrhr siRNA
14.95
6.12
PE-IL17A
FITC-CD4
P=0.0007
***
% IL-17A
Control
siRNA
Control siRNA
Ghrhr siRNA
Cell count (*1000)
FITC-GHRH-R
P=0.0211
*
MFI
l
IL-6 + TGF-β
DMSO
MR-409
MIA-602
20.71
0.11
0.22
30.31
0.13
0.14
12.47
0.07
0.25
11.62
0.06
0.27
13.87
0.04
0.20
11.23
0.03
0.24
PE-IL-17A
APC-IFN-γ
P<0.0001
****
% IL-17A⁺ IFN-γ⁺
% IFN-γ⁺
WT + DMSO
Ghrhr lit/lit + DMSO
WT + MR-409
Ghrhr lit/lit + MR-409
WT + MIA-602
Ghrhr lit/lit +MIA-602
m
IL-6 + TGF-β + IL-23
25.94
0.16
0.79
32.88
0.07
0.21
13.34
0.06
0.13
13.54
0.03
0.16
12.78
0.03
0.03
11.26
0.03
0.11
P=0.0003
***
n
IL-6 + TGF-β
IL-6 + TGF-β + IL-23
P=0.0159
Il10 relative mRNA expression
WT
Ghrhr lit/lit

Supplementary Fig. 5. GHRH-R signaling influences differentiation into Th17 cells but not T cell activation

(a-c) Naïve $CD4^+$ T cells isolated from WT and *Ghrhr*$^{lit/lit}$ mice were cultured under Th17 polarizing condition with various concentrations of hGHRH, MR-409 or MIA-602 for 3 days. IL-17A expression in the $CD4^+$ T cells were assessed by flow cytometry (n=2). (d-e) Naïve $CD4^+$ T cells isolated from WT and *Ghrhr*$^{lit/lit}$ mice were cultured under Th1 or Treg differentiation conditions with DMSO, MR-409 or MIA-602 for 3 days and analyzed for expression of IFN-γ, IL-4 and Foxp3 respectively by flow cytometry (n=3). (f-j) Naïve $CD4^+$ T cells were stimulated in Th17 cell conditions with or without MR-409 for 3 days for flow cytometry analysis of Ki67, live/dead cell dye 7-AAD and Annexin V, CD44, CD25, CD69 expression in the $CD4^+$ T cells (n=6). (g) The F value of one-way ANOVA test is 239.3, and the corresponding p-value is less than 0.0001. The exact p values (WT+DMSO vs WT+Rux, and WT+DMSO vs WT+Stattic) were 1×10^{-15} and 1×10^{-15} respectively. (k) Naïve T cells from WT mice were treated with control siRNA or *Ghrhr* siRNA and then cultured under Th17 cell-polarizing condition for 3 days. IL-17A and GHRH-R expression in the $CD4^+$ T cells were assessed by flow cytometry (n=6). Naïve $CD4^+$ T cells isolated from WT and *Ghrhr*$^{lit/lit}$ mice were cultured under (l) non-pathogenic (IL-6 and TGF-β) or (m) pathogenic (IL-6, TGF-β and IL-23) Th17 polarizing condition with DMSO, MR-409 or MIA-602 for 3 days. IL-17A and IFN-γ expression in the $CD4^+$ T cells were assessed by flow cytometry (n=6). The F values of one-way ANOVA test are 55.97 (l) and 63.32 (m), and the corresponding p-values are all less than 0.0001. (l) The exact p values (IL-$17A^+$ WT+DMSO vs *Ghrhr*$^{lit/lit}$+DMSO, IL-$17A^+$ WT+MR-409 vs *Ghrhr*$^{lit/lit}$+MR-409 and IL-$17A^+$ WT+DMSO vs WT+MIA-602) were 1.89×10^{-6}, 2.75×10^{-12} and 8.83×10^{-5} respectively. (m) The exact p values (IL-$17A^+$ WT+DMSO vs *Ghrhr*$^{lit/lit}$+DMSO, IL-$17A^+$ WT+MR-409 vs *Ghrhr*$^{lit/lit}$+MR-409 and

IL-17A^{+} WT+DMSO vs WT+MIA-602) were 2.68 x 10^{-8}, 1.45 x 10^{-12} and 1.87 x 10^{-6} respectively. (n) Gene expression of *Il10* was normalized to *Gapdh* and fold change is relative to the expression level of WT (n=5). Data are representation of at least two independent experiments. Data are presented as mean ± SEM. Two-sided student's t-test with Bonferroni correction (k and n) and one-way ANOVA followed by Bonferroni post hoc test (d-e, g-j, and l-m). *, **, *** and **** represent P<0.05, P̃<0.01, P̃ or (P)<0.001 and P̃<0.0001 respectively. ns represents no significant difference.

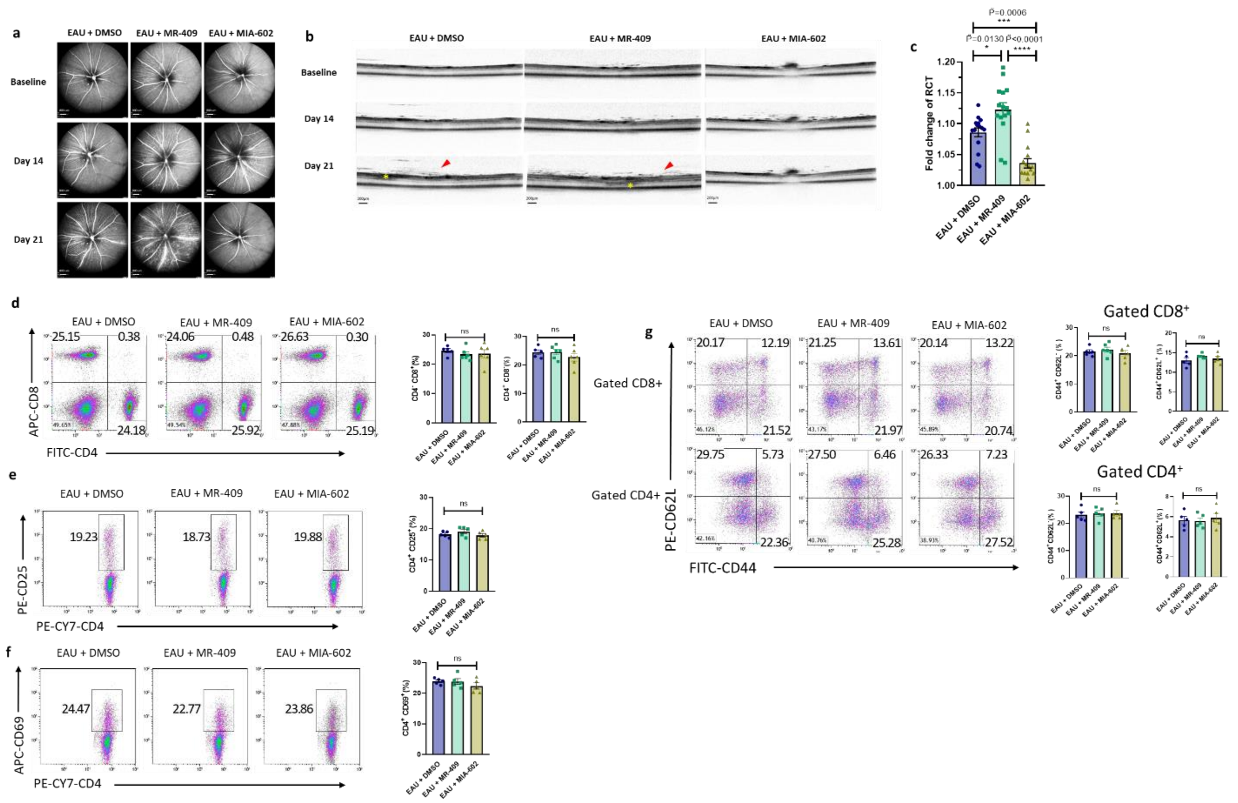

Supplementary Fig. 6. GHRH signaling does not alter lymphocyte population in the autoimmune ocular inflammation

(a-g) C57BL/6 WT mice were immunized with $IRBP_{1-20}$ peptide to induce EAU. From day 10-14 after immunization, mice were treated with DMSO, MR-409 or MIA-602 by subcutaneous injection daily to the end point (n=15). (a) Representative fundus images by cSLO at 3 time points after immunization. Scale bar: 800 μm. This experiment was repeated two times independently with similar results. (b) Representative images by OCT at 3 time points after immunization. Eye-infiltrating cells (arrow heads) and retinal folds (asterisks) in the vitreous and retina are indicated. Scale bar: 200 μm. This experiment was repeated two times independently with similar results. (c) Quantified fold change of retinal choroidal thickness assessed by OCT at day 21 after immunization (n=15). The F value of one-way ANOVA test is 24.80, and the corresponding p-value is less than 0.0001. The exact p value (EAU+MR-409 vs EAU+MIA-602) was 4.15×10^{-8}.

(d-g) At day 21 post-immunization, cells were isolated from the eye-draining lymph nodes (n=10). (d) Flow cytometry analysis of CD4 and CD8 population in the lymphocytes (n=6). (e-f) Flow cytometry analysis of CD25 and CD69 expression in the $CD4^+$ T cells (n=5). (g) CD44 and CD62L expression in the $CD4^+$ and $CD8^+$ T cells (n=5). Data are presented as mean ± SEM. P-values were all determined by one-way ANOVA followed by Bonferroni post hoc test. *, *** and **** represent $\widetilde{P}<0.05$, $\widetilde{P}<0.001$ and $\widetilde{P}<0.0001$ respectively. ns represents no significant difference.

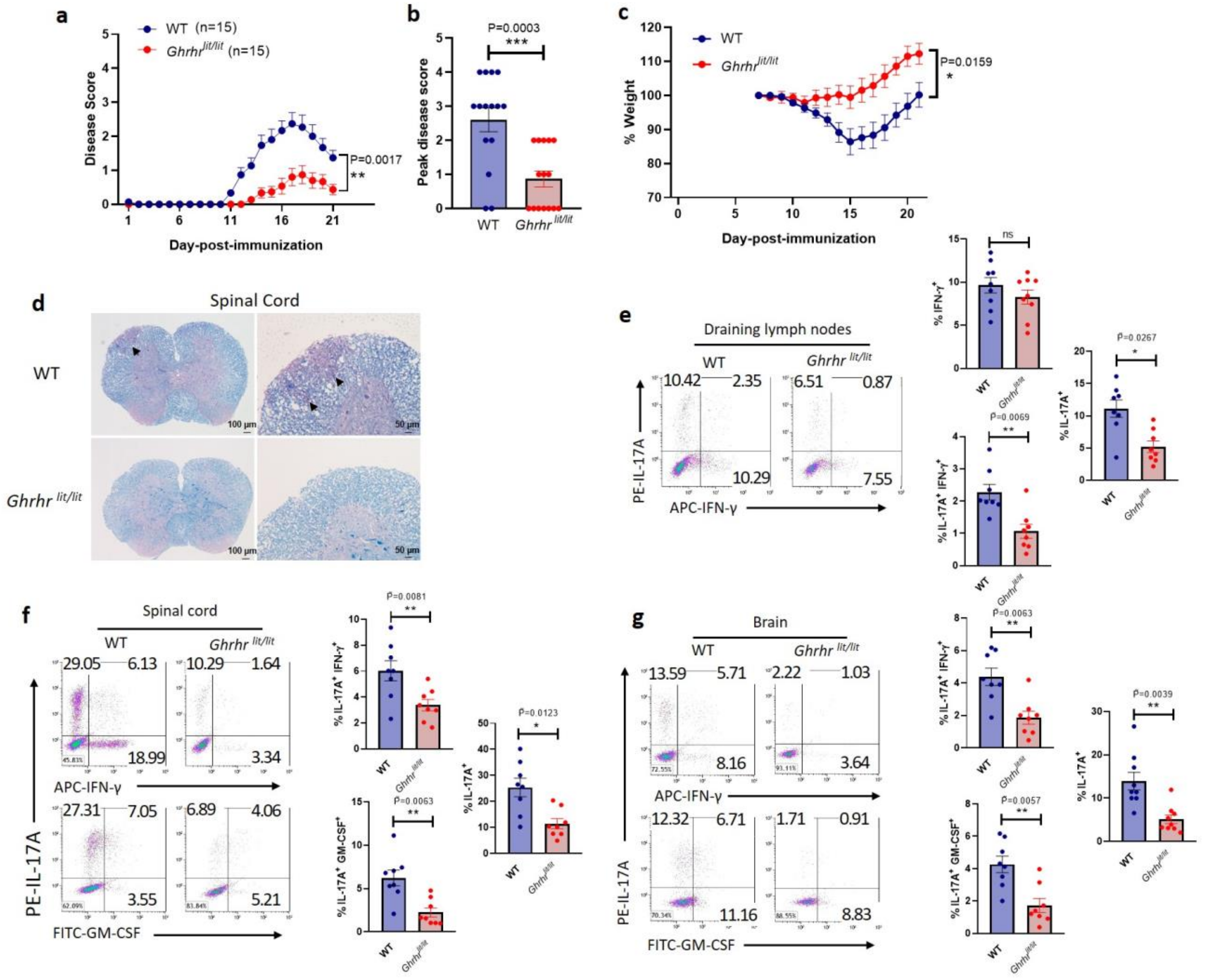

Supplementary Fig. 7. Deficiency of GHRH-R protects mice from Th17-mediated autoimmune neuroinflammation

WT and *Ghrhr*$^{lit/lit}$ mice were challenged with EAE. After 21 days, eyeballs were collected for Luxol fast blue-staining (n=15). (a) Disease score throughout 21 days and (b) the peak disease score. (c) Fold change of daily body weight. (d) CNS-infiltration (arrows) in the sections of spinal cord (n=5). Scale bars: 100 µm (left) and 50 µm (right). (e-g) Cells were isolated from draining lymph nodes for flow cytometry analysis of IFN-γ, IL-17A and GM-CSF expression in CD4+ T cells from spleen, spinal cord and brain (n=8). Data are representation of at least two independent experiments. Data are presented as mean ± SEM. Student's t-test (a-c) and Student's t-test with

Bonferroni correction (e-g). Statistical tests were all two-sided. *, **, *** and **** represents $\widetilde{P}$ (or P)<0.05, $\widetilde{P}$ (or P)<0.01 and P<0.01 and p<0.001 respectively. ns represents no significant difference.
