# Supplementary material for: Growth hormone releasing hormone signaling promotes Th17 cell differentiation and autoimmune inflammation
Source: Nat Commun. 2023 Jun 6;14:3298. doi: 10.1038/s41467-023-39023-1 (PMC10244428; doi:10.1038/s41467-023-39023-1)
Supplement: Supplementary file 3 — Description of Additional Supplementary Files [file 41467_2023_39023_MOESM3_ESM.pdf]

Description of Additional Supplementary Files

Supplementary Data 1: This file provides information about the reagents and computer software used in this study.
