# Supplementary material for: Growth hormone releasing hormone signaling promotes Th17 cell differentiation and autoimmune inflammation
Source: Nat Commun. 2023 Jun 6;14:3298. doi: 10.1038/s41467-023-39023-1 (PMC10244428; doi:10.1038/s41467-023-39023-1)
Supplement: Supplementary file 5 — Reporting Summary [file 41467_2023_39023_MOESM5_ESM.pdf]

nature portfolio

Corresponding author(s): Wai Kit Chu

Last updated by author(s): May 2, 2023

Reporting Summary

Nature Portfolio wishes to improve the reproducibility of the work that we publish. This form provides structure for consistency and transparency in reporting. For further information on Nature Portfolio policies, see our Editorial Policies and the Editorial Policy Checklist.

Statistics

For all statistical analyses, confirm that the following items are present in the figure legend, table legend, main text, or Methods section.

n/a	Confirmed	
[ ]	[x]	The exact sample size (*n*) for each experimental group/condition, given as a discrete number and unit of measurement
[ ]	[x]	A statement on whether measurements were taken from distinct samples or whether the same sample was measured repeatedly
[ ]	[x]	The statistical test(s) used AND whether they are one- or two-sided<br>*Only common tests should be described solely by name; describe more complex techniques in the Methods section.*
[x]	[ ]	A description of all covariates tested
[x]	[ ]	A description of any assumptions or corrections, such as tests of normality and adjustment for multiple comparisons
[ ]	[x]	A full description of the statistical parameters including central tendency (e.g. means) or other basic estimates (e.g. regression coefficient) AND variation (e.g. standard deviation) or associated estimates of uncertainty (e.g. confidence intervals)
[ ]	[x]	For null hypothesis testing, the test statistic (e.g. *F*, *t*, *r*) with confidence intervals, effect sizes, degrees of freedom and *P* value noted<br>*Give P values as exact values whenever suitable.*
[x]	[ ]	For Bayesian analysis, information on the choice of priors and Markov chain Monte Carlo settings
[x]	[ ]	For hierarchical and complex designs, identification of the appropriate level for tests and full reporting of outcomes
[x]	[ ]	Estimates of effect sizes (e.g. Cohen's *d*, Pearson's *r*), indicating how they were calculated

Our web collection on statistics for biologists contains articles on many of the points above.

Software and code

Policy information about availability of computer code

Data collection	Cytomics FC500 Flow Cytometry (Beckman), MoFlo Astrios (Beckman) or LSRII (BD Biosciences)
Data analysis	Kaluza 2.1, FlowJo 10.8.1, Image J 1.45d, NIS-Elements 5.21.00

For manuscripts utilizing custom algorithms or software that are central to the research but not yet described in published literature, software must be made available to editors and reviewers. We strongly encourage code deposition in a community repository (e.g. GitHub). See the Nature Portfolio guidelines for submitting code & software for further information.

Data

Policy information about availability of data

All manuscripts must include a data availability statement. This statement should provide the following information, where applicable:

- Accession codes, unique identifiers, or web links for publicly available datasets
- A description of any restrictions on data availability
- For clinical datasets or third party data, please ensure that the statement adheres to our policy

All raw data is provided in the Source Data file.

Human research participants

Policy information about studies involving human research participants and Sex and Gender in Research.

Reporting on sex and gender	N/A
Population characteristics	N/A
Recruitment	N/A
Ethics oversight	N/A

Note that full information on the approval of the study protocol must also be provided in the manuscript.

Field-specific reporting

Please select the one below that is the best fit for your research. If you are not sure, read the appropriate sections before making your selection.

- [x] Life sciences
- [ ] Behavioural & social sciences
- [ ] Ecological, evolutionary & environmental sciences

For a reference copy of the document with all sections, see nature.com/documents/nr-reporting-summary-flat.pdf

Life sciences study design

All studies must disclose on these points even when the disclosure is negative.

Sample size	Sample size was choose based our previous publication (Liang et al. PNAS 117, p6067, 2020).
Data exclusions	No data were excluded for analysis.
Replication	At least 2 different biologically independent samples were studied to show consistent results. All replications were successful.
Randomization	Animals were allocated into experimental groups randomly.
Blinding	No blinding was applied during data collection or analysis. The Ghrhr lit/lit mice had obvious smaller body size than the wild type mice. Similarly, the MIA-602 treated mice had obvious less inflammation. Therefore blinding was not possible.

Reporting for specific materials, systems and methods

We require information from authors about some types of materials, experimental systems and methods used in many studies. Here, indicate whether each material, system or method listed is relevant to your study. If you are not sure if a list item applies to your research, read the appropriate section before selecting a response.

Materials & experimental systems

n/a	Involved in the study	
[ ]	[x]	Antibodies
[x]	[ ]	Eukaryotic cell lines
[x]	[ ]	Palaeontology and archaeology
[ ]	[x]	Animals and other organisms
[x]	[ ]	Clinical data
[ ]	[ ]	Dual use research of concern

Methods

n/a	Involved in the study	
[x]	[ ]	ChIP-seq
[ ]	[x]	Flow cytometry
[x]	[ ]	MRI-based neuroimaging

Antibodies

Antibodies used	Purified anti-mouse CD3 Antibody (145-2C11) BioLegend 100302<br>Purified anti-mouse CD28 Antibody (37.51) BioLegend 102102<br>Purified anti-mouse IL-4 Antibody (11B11) BioLegend 504102<br>Purified anti-mouse IFN-γ Antibody (XMG1.2) BioLegend 505802<br>PerCP anti-mouse CD3ε Antibody (145-2C11) BioLegend 100326<br>FITC anti-mouse CD4 Antibody (RM4-5) BioLegend 100510<br>PE/Cyanine7 anti-mouse CD4 Antibody (GK1.5) BioLegend 100422

PE anti-mouse IL-17A Antibody (TC11-18H10.1) BioLegend 506904
APC anti-mouse IFN-γ Antibody (XMG1.2) BioLegend 505810
PE anti-mouse CD25 Antibody (PC61) BioLegend 102008
APC anti-mouse CD196 (CCR6) Antibody (29-2L17) BioLegend 129814
APC anti-mouse CD69 Antibody (H1.2F3) BioLegend 104514
PE Hamster Anti-Mouse CD69 BD 553237
PE anti-mouse IL-4 Antibody (11B11) BioLegend 504104
Alexa Fluor® 647 anti-mouse FOXP3 Antibody (MF-14) BioLegend 126408
Alexa Fluor® 488 anti-mouse FOXP3 Antibody (MF-14) BioLegend 126406
FITC anti-mouse GM-CSF Antibody (MP1-22E9) BioLegend 505404
PE anti-GATA3 Antibody (16E10A23) BioLegend 653804
FITC anti-T-bet Antibody (4B10) BioLegend 644812
APC anti-mouse CD62L Antibody (MEL-14) BioLegend 104412
PE anti-mouse CD62L Antibody (MEL-14) BioLegend 104408
FITC anti-mouse/human CD44 Antibody (IM7) BioLegend 103006
PE/Cyanine7 anti-mouse CD8a Antibody (53-6.7) BioLegend 100722
APC anti-mouse CD8a Antibody (53-6.7) BioLegend 100712
Alexa Fluor® 488 Mouse Anti-Stat3 (pY705) (4/P-STAT3) BD 557814
Alexa Fluor® 647 Mouse Anti-Mouse RORγt (Q31-378) BD 562682
PE Mouse anti-Mouse RORγt (Q31-378) BD 562607
Alexa Fluor® 647 Rat anti-Mouse Foxp3 (MF23) BD 560401
Phospho-Stat3 (Tyr705) (D3A7) Rabbit CSL 9145S
Stat3 (124H6) Mouse CSL 9139S
Anti-IGF-I Antibody (H-9) Santa Cruz sc-518040
Anti-GH Antibody (E-7) Santa Cruz sc-374266
Anti-GHR Antibody (B-10) Santa Cruz sc-137185
Anti-IGF-1 Receptor α/IGF1R Antibody (1H7) Santa Cruz sc-461
Anti-GHRHR antibody Abcam ab76263
Anti-GHRH antibody Abcam ab187512
Purified anti-mouse CD16/32 Antibody (93) BioLegend 101302

The dilutions or exact concentrations of these antibodies are stated in the Supplementary Table.

Validation

All antibodies were obtained from commercial sources. Validation statements can be found on the manufacturers' websites.

Animals and other research organisms

Policy information about studies involving animals; ARRIVE guidelines recommended for reporting animal research, and Sex and Gender in Research

Laboratory animals

Wild type mice (C57BL/6J, Jax 000664) and Ghrhrlit/lit mice (C57BL/6J-Ghrhrlit/J, Jax 000533) were purchased from The Jackson Laboratories. 6-10 weeks old animals were used.

Wild animals

This study did not involve wild animals.

Reporting on sex

Both male and female mice were used. Sex was not analyzed as there is no publication reported GHRH-R could regulate Th17 differentiation in a sex dependent manner.

Field-collected samples

This study did not involve field-collected samples.

Ethics oversight

Ethics approval for this study was obtained from the Animal Experimentation Ethics Committee of the Chinese University of Hong Kong (approval numbers 20/014/GRF and 22/270/MIS).

Note that full information on the approval of the study protocol must also be provided in the manuscript.

Dual use research of concern

Policy information about dual use research of concern

Hazards

Could the accidental, deliberate or reckless misuse of agents or technologies generated in the work, or the application of information presented in the manuscript, pose a threat to:

No	Yes	
☒	☐	Public health
☒	☐	National security
☒	☐	Crops and/or livestock
☒	☐	Ecosystems
☒	☐	Any other significant area

Experiments of concern

Does the work involve any of these experiments of concern:

No	Yes	
☒	☐	Demonstrate how to render a vaccine ineffective
☒	☐	Confer resistance to therapeutically useful antibiotics or antiviral agents
☒	☐	Enhance the virulence of a pathogen or render a nonpathogen virulent
☒	☐	Increase transmissibility of a pathogen
☒	☐	Alter the host range of a pathogen
☒	☐	Enable evasion of diagnostic/detection modalities
☒	☐	Enable the weaponization of a biological agent or toxin
☒	☐	Any other potentially harmful combination of experiments and agents

Flow Cytometry

Plots

Confirm that:

☒ The axis labels state the marker and fluorochrome used (e.g. CD4-FITC).

☒ The axis scales are clearly visible. Include numbers along axes only for bottom left plot of group (a 'group' is an analysis of identical markers).

☒ All plots are contour plots with outliers or pseudocolor plots.

☒ A numerical value for number of cells or percentage (with statistics) is provided.

Methodology

Sample preparation	Mice were sacrificed prior to isolation of spleen, lymph nodes, eyes or CNS tissues. Secondary lymphoid tissues were collected and grinded into suspension using a syringe and a 70 μm strainer. Red blood cells were lysed using RBC lysis buffer (BioLegend). Remaining cells were washed, filtered with 40 μm strainer and resuspended in complete RPMI (RPMI medium containing 10% fetal bovine serum, 100 IU/ml penicillin, 100 μg/ml streptomycin, 1 mM sodium pyruvate, nonessential amino acids and 55 μM β-mercaptoethanol) for further experiments. For EAU experiments, eyes were collected from mice induced with EAU 19-21 days after immunization. After removal of extraocular tissues, the lens was enucleated from eyes and the remaining tissue was minced into small pieces with scissors and incubated in complete RPMI medium containing 1 mg/ml of collagenase D and 1 mg/ml of DNase (QIAGEN) for 45 minutes at 37°C. Tissues were then dispersed vigorously by pipetting several times, filtered with 70 μm strainer, and re-stimulated in the complete RPMI supplemented with 50 ng/ml phorbol myristate acetate (PMA, Sigma), 500 ng/ml ionomycin (Sigma) and 5 μg/ml brefeldin A (BioLegend) for 4 hours prior to intracellular staining for flow cytometry. For EAE experiments, spinal cords and brains were collected from mice induced with EAE 21 days after immunization. These tissues were minced into small pieces and incubated in the complete RPMI containing 1mg/ml of collagenase D and 1 mg/ml of DNase for 45 minutes at 37°C. Then the samples were disrupted by pipetting several times, strained over a 70 μm filter and purified by centrifugation with Percoll gradient at 13,000 g at 4°C for 10 minutes. After washed, cells were resuspended in complete RPMI containing 50 ng/ml PMA (Sigma), 500 ng/ml ionomycin (Sigma) and 5 μg/ml brefeldin A (BioLegend) for 4 hours prior to intracellular staining for flow cytometry.
Instrument	Cytomics FC500 Flow Cytometry (Beckman)
Software	Kaluza, FlowJo

Cell population abundance	This study did not involve cell sorting.
Gating strategy	All gating strategy was described in Supplementary Fig. 4.

☒ Tick this box to confirm that a figure exemplifying the gating strategy is provided in the Supplementary Information.
